# The Long and Winding Road: A Caregiver’s Journey

**DOI:** 10.1093/geroni/igab046.1186

**Published:** 2021-12-17

**Authors:** Dolores Gallagher-Thompson, Theresa Harvath

**Affiliations:** 1 Stanford University School of Medicine, LOS ALTOS, California, United States; 2 University of California, Davis, UC Davis, California, United States

## Abstract

In a discussion about innovative caregiving programs, it is important to hear the voice of the caregiver. In this section, using her personal experience, this healthcare professional will explore her interactions with the medical system as a caregiver for her partner. She will discuss her caregiver journey from the cancer diagnosis in 2014 through her partner’s final days in hospice, which occurred during the pandemic. She will discuss pertinent issues regarding in what ways the healthcare system response was helpful, which responses were problematic, and where it outright failed to address caregiver needs. Issues, such as caregiver perceived invisibility during the hospital stay, how ageism affected policy decisions and what effect those policies had on her as the caregiver, and other important issues will be explored, including the how the pandemic affected her as a caregiver.

